# Supplementary figures and images for: Inhibition of miR-199b-5p reduces pathological alterations in osteoarthritis by potentially targeting Fzd6 and Gcnt2
Source: eLife. 2024 May 21;12:RP92645. doi: 10.7554/eLife.92645 (PMC11108644; doi:10.7554/eLife.92645)

CD9

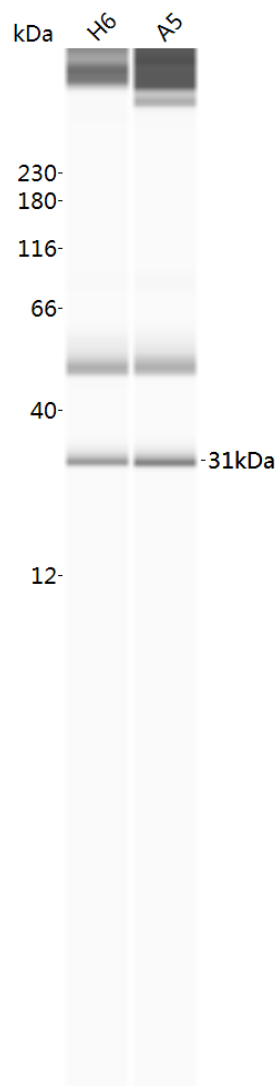

CD63

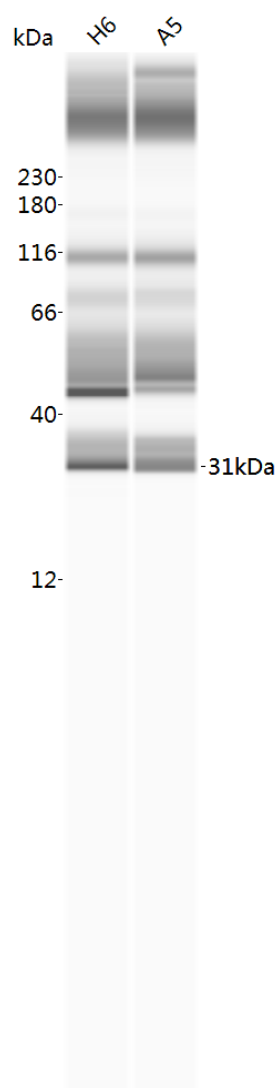

CD81

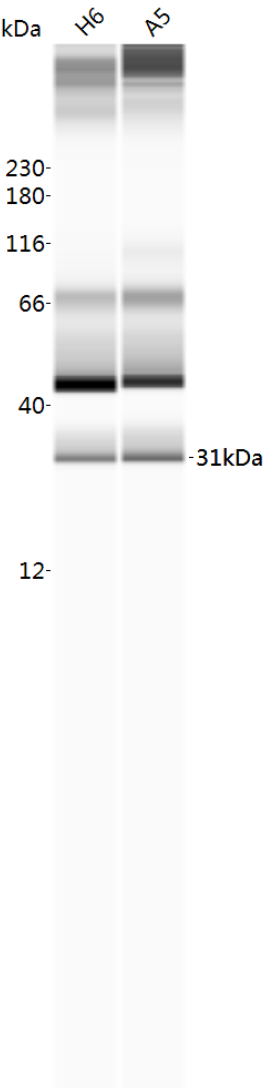

Supplement: Figure 1—figure supplement 1—source data 1. [file elife-92645-fig1-figsupp1-data1.pdf]

CD9

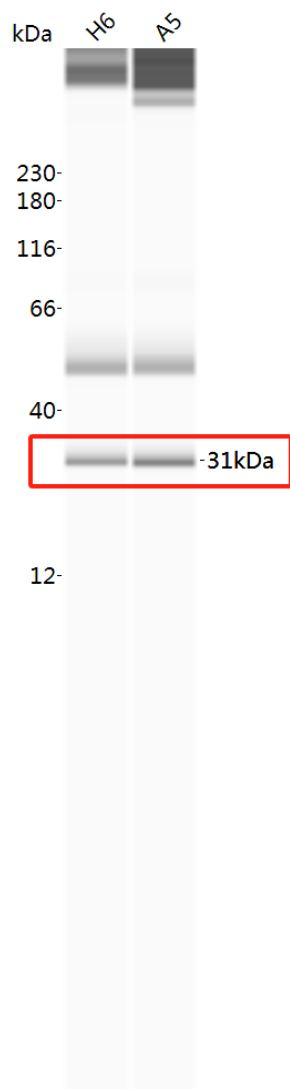

CD63

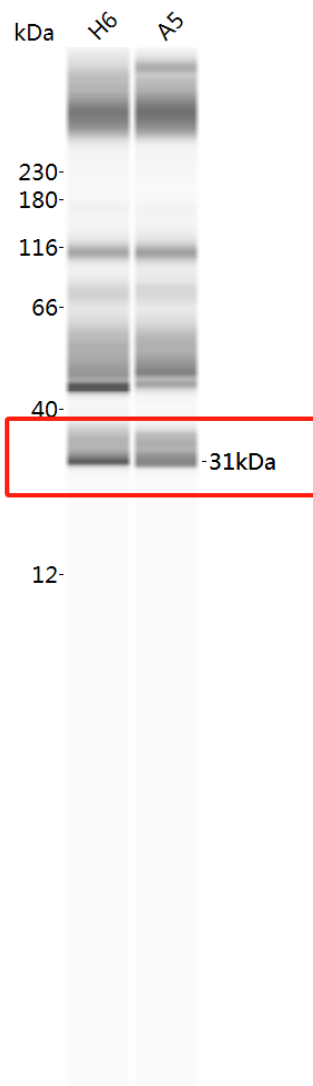

CD81

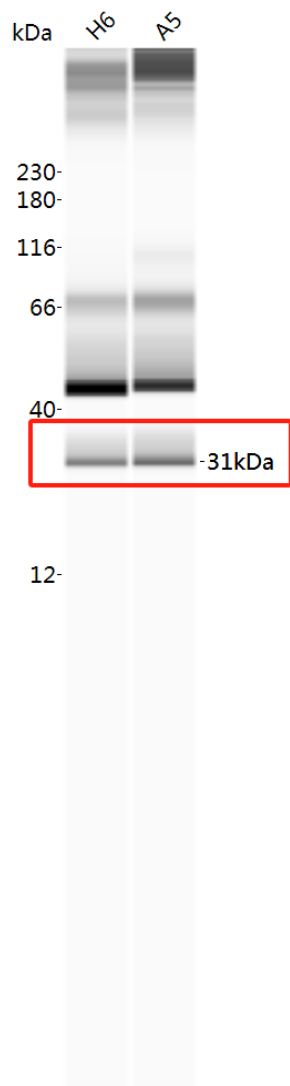

Supplement: Figure 1—figure supplement 1—source data 2. [file elife-92645-fig1-figsupp1-data2.pdf]
