# Supplementary material for: Inhibition of miR-199b-5p reduces pathological alterations in osteoarthritis by potentially targeting Fzd6 and Gcnt2
Source: eLife. 2024 May 21;12:RP92645. doi: 10.7554/eLife.92645 (PMC11108644; doi:10.7554/eLife.92645)
Supplement: Figure 6—source data 2. [file elife-92645-fig6-data2.docx]

>mmu-miR-199b-5p MIMAT0000672

CCCAGUGUUUAGACUACCUGUUC

UGUGACC ACACTGG

UGUGAC ACACTG

GUGACC CACTGG

Mus musculus frizzled class receptor 6 (Fzd6), transcript variant 1, mRNA

NCBI Reference Sequence: NM_008056.3

AGAAAACTGTCTCGTTCCCCCAGAAGCACATGTATGTTACACTGGAGATGACCAACTGATTTGTCTTATAAAGGCCACTGTTGAGCTGGGAGAGTAGCCCAGTGGTACAGCGCCCACCTGGAATACTTGAGGACCTGGGGTTGTCTCCCAGCACTGCAAAAGGAAAATTCACTGTTACAGTCTTCCTTGCACTTAAACCAGCTTTGTCTATTGTTTTTTTGGTTTGGCTTTTATTTTTGTTGCTGTTATTTTTGTTGTTGTTTGTTTGTTTTTTTGTTTGTTTGTTTGAGACAGGGTTTCTTTGCTAGCCCTGACTGTCCTGAAACTCCCTCTGTAGACCAGGCTGGCCTCAAACTTACAGAGATCTGCCTGCCTCAGCCTCCCGAGTGCTGGGAATAATGGTGTGGTCACCACTGCCCAGCCTTTTGTCTGTTTTTAAACTTGAAAGAAACAACAGCCCAGATTTCAAAAATAATATAATGCATTTATACCTAAAAAACCAACCAGGAGTGCCCAGTTAATAACACTTTTTAAATGTGGGGATGGGAAGGGCATTAGAGGAGTCTTCCTTCTATTGAAGATTCATTAAAGTATTTTAAGATATGCTCTTTCACTCTTTATATAAATCCAAGATTTTTCTTTGCTGAAGTATTTAAAACTTTTGTACCTTTATATGTAGATATGAATTTGAAAATATGCTTATGTGTATTTGAACTTTTGAAAATCCTAGAGAATTGAATCAAATATTTTTATGATGTTTTTCTACTATTTTAGCTACTTTGCGACTGTGATAGCTGTTACACTGGATTTTTAAAAAACTTGTACAGCAGCCTCTTTACAGTAAAAAGAGTGGGTGTCACACTGAAAGGTCTGTAAGAAGTGGTCACAGCCACCCCTACCTTCCCCAAAAGGAGGAACTTGGTGGCAGGTCCCTCCCTGATTGGACTGTCCCTTTCTTTCTGCATGTTATAAATCAGCAGGTAAGATGGTAGGTTTTTACAAGTTAGGCCGAGCTGTCGATTCCCCTTTTAAGTGTTGAATTAGGATTGAATTATGGCCATTTGTAGTTGCTCGTGCCTGTCTTTATTTTAGTATTTTATTTCCCGAGACAGGAACTCACTGTGTGGTGCTCCTTGGCTGTCTGGTGTTCAGTCTGTCCCAGGCAGGTCACAGAGATCTCCCCCTCTGCAGCCCACTCATCTCTCCCAAGCCACCACACTCAGCTTTTATCTGTTTTAAAAATTTAAACTTAAAAAAATGTTTTTGGAATAGTACAAACACATTGTGTTGTAAATTTCTTTGATGCTATGCAAAATTCCTATCTGCATCTAAGCCTGCAAAAGAAAATGTGCGAAGGGCAGAGTCAGAGTTGGGCAGGAAGAGTGTAGTGCAGCAGATGCAGCGTGAAGACACTGAAGGTGCTAAGACAGCGTCTCAGTGCTGGTCCTCCTTAAGGATTATCTCGCCAGCGAGGTTTTCTTAGATACTTTGATCCCATTGGAGCTCTGTTAAAGTTTAAAATGAAAATTATCATGTACTGTATGGGAAATGTAAATACTAACTTTTCCACATATGTAAACTTCAGACACAAATTTTTTTGTGTGTTCTTTTCATCAATAAAATTTTCTTTGTATAAAAAAA

拼接序列：

AGAAAACTGTCTCGTTCCCCCAGAAGCACATGTATGTTACACTGGAGATGACCAACTGATTTGTCTTATAAAGGCCACTGTTGAGCTGGGAGAGTAGCCCAGTGGTACAGCGCCCACCTGGAATACTTGAGGACCTGGGGTTGAATTGAATCAAATATTTTTATGATGTTTTTCTACTATTTTAGCTACTTTGCGACTGTGATAGCTGTTACACTGGATTTTTAAAAAACTTGTACAGCAGCCTCTTTACAGTAAAAAGAGTGGGTGTCACACTGAAAGGTCTGTAAGAAGTGGTCACAGCCACCCCTACCTTCCCCAAAAGGAGGAACTTGGTGGCAGGTCCCTCCCTGATTGGACTGTCCTATCTGCATCTAAGCCTGCAAAAGAAAATGTGCGAAGGGCAGAGTCAGAGTTGGGCAGGAAGAGTGTAGTGCAGCAGATGCAGCGTGAAGACACTGAAGGTGCTAAGACAGCGTCTCAGTGCTGGTCCTCCTTAAGGA

>hsa-miR-199b-5p MIMAT0000263

CCCAGUGUUUAGACUAUCUGUUC

NCBI Gene ID 8323 GenBank Accession NM_001164615

Gene Symbol FZD6 3' UTR Length 1368

Gene Description frizzled class receptor 6

3' UTR Sequence ：agaacattttctctcgttactcagaagcaaatttgtgttacactggaagtgacctatgcactgttttgtaagaatcactgttacattcttcttttgcacttaaagttgcattgcctactgttatactggaaaaaatagagttcaagaataatatgactcatttcacacaaaggttaatgacaacaatatacctgaaaacagaaatgtgcaggttaataatatttttttaatagtgtgggaggacagagttagaggaatcttccttttctatttatgaagattctactcttggtaagagtattttaagatgtactatgctattttacttttttgatataaaatcaagatatttctttgctgaagtatttaaatcttatccttgtatctttttatacatatttgaaaataagcttatatgtatttgaacttttttgaaatcctattcaagtatttttatcatgctattgtgatattttagcactttggtagcttttacactgaatttctaagaaaattgtaaaatagtcttcttttatactgtaaaaaaagatataccaaaaagtcttataataggaatttaactttaaaaacccacttattgataccttaccatctaaaatgtgtgatttttatagtctcgttttaggaatttcacagatctaaattatgtaactgaaataaggtgcttactcaaagagtgtccactattgattgtattatgctgctcactgatccttctgcatatttaaaataaaatgtcctaaagggttagtagacaaaatgttagtcttttgtatattaggccaagtgcaattgacttcccttttttaatgtttcatgaccacccattgattgtattataaccacttacagttgcttatattttttgttttaacttttgttttttaacatttagaatattacattttgtattatacagtacctttctcagacattttgtagaattcatttcggcagctcactaggattttgctgaacattaaaaagtgtgatagcgatattagtgccaatcaaatggaaaaaaggtagttttaataaacaagacacaacgtttttatacaacatactttaaaatattaaggagttttcttaattttgtttcctattaagtattattctttgggcaagattttctgatgcttttgattttctctcaatttagcatttgcttttggtttttttctctatttagcattctgttaaggcacaaaaactatgtactgtatgggaaatgttgtaaatattaccttttccacattttaaacagacaactttgaatacaaaaactttgttttgtgtgatcttttcattaataaaattatctttgtataagaaaaaaaaaaaaaa

>mmu-miR-199b-5p MIMAT0000672

CCCAGUGUUUAGACUACCUGUUC

UGUGACC ACACTGG

UGUGAC ACACTG

GUGACC CACTGG

Mus musculus glucosaminyl (N-acetyl) transferase 2, I-branching enzyme (Gcnt2), transcript variant 1, mRNA

NCBI Reference Sequence: NM_008105.3

CCCGCAGCAGCTCGGGCCTAAATGGAAATTGAAGACGTAAAGAAGAGCTTTCTTTTCCAAGAGACTCTGGTCTTGGCTATGCTGAAGACTTTTTTAAAAAATGGTTTTCAGGGAACCGTGAGGATCTGGCAACATGGCTCTGCTTGCAATATCCACTGAGCACTGTAATACATTTGACAGGATGGCTGCATGAAACTGTCCAGCATTTTTCTGTCGTTTCCCCCCCACCCCAAATCCTTTTTTTCTCTTCTTTTTTAAAAGATAGGTAGGGTCTTGCTAGATATCTCTAGCAAGCCTGTTTCTTGTCAGGTAGCCCAGGATGGCCTTGAACTCTTAATCCTCCGCCTTGGCCCTCTAAGGGCTGCTAGTCCGAGTGTGGACAACCACACCTGGCTCTGGCGTCTGGCGGTCACTTGACGCTCATCTGCGTCTCCGTCTGCTAGGTGGTCGGTGATAAATTCAAATGTATCAGATCTCTGTGCCAGATACTCATATTAGTATACCCTGTTCAAAGGAGAATGGTGGATAATACTGTCTGAGGAAATCAGAACTTATTTGAGAAGGAAGCTCTCGTTCCACATTCCTAGCAACTATATTCTCAGGCACACATAGGCGCACAGAGACAGCTTCCTAGTAACTCACATCAATTTCGTTTAATACAAACTGATCCTGAAGAGCTGAGGAACTATGTAAGTTCAGTGTCCAAGCATTTGACTGACATTTGTGCAGAGTTAGGTTCCATCTCCAGCATCACGCACGGGTCTTGTGAACACCGACATCTTCAGTTTACTTGTGTCTGCAGTTCTGTGGACAGAACAAAGCCATTCACAAGTGACCGACAGCACGTGCCGTGAGACGGGAGGTGGCTGCAGGAAGAGGATGCCTCAGGGATGCCGGACATTTCCGTGTTCTATTCCAGAGTGTTCTCTTCTCCCAGAGAACACTGGAACGTCCTCACAGGGATGAAGGAATTCTTTCTTTCCCACAGGAAAGGTGCTACTGTGTGGGTCTGGAACCTGTGAATGAATCACTCCTGGGGTTTCGTTTCTATGTGAGAGACGGGGACCCGGTTCTGTTGTGTGCAGCTTTAGCGGCATCAGGACAGCTTCCAGCTTGCAATGATTGCTGCTGACAGGAACACCTGGATCTCAAGAGCTTTTTATGACAGGTGGACATCTGTGTCATTTTTCCTTGTTCCCTCATCCATCCATCCATCCATCCATCCATCCATCCATCCATCCATCCATTCGTCAGCCTACATTCAACAAACGCCTCTTGAATGTCTATGATTCCCCCAGACACATGCTGACAGACACGAGGGAAGCAAGACAGATTCTTTCTCCACTGTTAAACTCTGGGGAGTGGTATCAGCTTTTGAAACCCTGCTCTAGACTCTTACAAAATGGCTACACTTAGCACATACTCTGCCCTGGCTATATATTATATTGGGCCATAGAGATCAGCAATGTATCAATTAATAGATTAATCCAGAGACAGGAAGGAGGAAACGAAAGCTAGAGCCTCTTTGTGACTCCACCAACTCCTTCTTGTCTCCTTTTATTGAAGAGTCCGCACACATACTCACCCCTCTGGTGGGCCACATGAGGACCTGCATCTTGCAGATGATGTAGCAAGCCTTAGGATGGTTCATGATCAGTAGGAAGCAAATTCTAAGACCTCAGGGTGTCTTTCTGAGTCTCTCATTTCTTTAGGGATGTGGAGAGAAGATGAAGTATAGCCGTGTGTAGTGTCGCCAGAGAAGGTGGAGTTTAAGAATGGTCTCAGTGTTATTAATGAGAAAAAGTTCTGTCATTCAGGAAAAAAAAGCAAAAAACGTGAGGTTGGGACTCAGTAGGTGAGGGTGCTTGCAGCCAAACCTGATGACCTGCATTCAATTCCTGGGTCCCACGGGACAGAGGGAGAGAGTCCACTACTGAAAGGAACGCTCAAACCTCCTCACACAGGTCCTTGTGCCTGGGCACTCCCCCGCCCCATATGCATACACACATACATGCTTTTGTGATTTTTTTTATTAGTGCTTTTACATTTAGAAATATTTATTTATTCTAGCAGGGAAAAAACCCCACATGCTTTAAATGATGTCACTTTCCACAACTTCATTCTTGTGCTGTCATTGGACTGAATGTTATGCATCTTTAAAGGGAATCACTTCGTGTAAAACTCTCTTGTCTCTTCTGCTGAAAAGTAAAATGGGTTGTGTCGGGTAATAAAGGGTCGAGGGGTAGGAAGCACTTGGTCAAGGCGAAGTCTAAAACCGGTCTCTTTTTTATTTGTTACTTGGATGAGAGCACCTGAAGACTAATGTTTTACACTGGATTGAGATCTCATGGAAAATGAAACATTCTTTTTCTGGAGAAGTTTACATGCCCATTGTTGGTCACCTCTTTACTGTAAATACTATTGAAAAAAGGAGCGTAGCATTTGCATAGCAGGGATTGTGCCTGTATCTACTGTGTCCATTTTGTTTTTTCGGATGTTTTCTGGTTTTGTTTCCTTGGAAATAAATTAATATATTGTTTTCCACCTTCAAAAAAAAAAAAAAAAAAA

拼接序列：

AGTTCTGTGGACAGAACAAAGCCATTCACAAGTGACCGACAGCACGTGCCGTGAGACGGGAGGTGGCTGCAGGAAGAGGATGCCTCAGGGATGCCGGACATTTCCGTGTTCTATTCCAGAGTGTTCTCTTCTCCCAGAGAACACTGGAACGTCCTCACAGGGATGAAGGAATTCTTTCTTTCCCACAGGAAAGGTGCTACTGTGTGGGTCTGGAAAACTCTCTTGTCTCTTCTGCTGAAAAGTAAAATGGGTTGTGTCGGGTAATAAAGGGTCGAGGGGTAGGAAGCACTTGGTCAAGGCGAAGTCTAAAACCGGTCTCTTTTTTATTTGTTACTTGGATGAGAGCACCTGAAGACTAATGTTTTACACTGGATTGAGATCTCATGGAAAATGAAACATTCTTTTTCTGGAGAAGTTTACATGCCCATTGTTGGTCACCTCTTTACTGTAAATACTATTGAAAAAAGGAGCGTAGCATTTGCATAGCAGGGATTGTGCCT

>hsa-miR-199b-5p MIMAT0000263

CCCAGUGUUUAGACUAUCUGUUC

NCBI Gene ID 2651 GenBank Accession NM_001491

Gene Symbol GCNT2 3' UTR Length 2780

Gene Description glucosaminyl (N-acetyl) transferase 2 (I blood group)

3' UTR Sequence:

gctattcatgagctactcatgactgaagggaaactgcagctgggaagaggagcctgtttttgtgagagacttttgccttcgtaatgttaaccgtttcaggaccacgtttatagcttcaggacctggctacgtaattatacttaaaatatccactggacactgtgaaatacactaacaggatggctgggtagagcaatctgggcactttggccaattttagtcttgctgtttcttgatgctcacctctatattagtttattgttaggatcaatgataaatttaaatgacctcagatctttgcaccagatactcatcatatacaaatgttttagtaaaaaagagaattgtagataatactgtctaggaaaataagaattaggtttctttgaagaaggaatcttttataacaccttaacagtcaccactgtgctcaaccagacagatagtgaaacagctttctgggtaattcaccaatttcctttaaaacataagctacctgaatggagaatacatcttgtttctgagtttcaacactagcatttttggcttactcatggacaaagttctgtatatagtataaagtcattaacaagaaacaggatatgctttaagacagaattcactgtctgttgcttcagtaaaaggacctcggggaataaaacatttctctcttatatgccagaatgtaggctggtccctatgtcatgtcttccattaagaacactaaaaagtccttgcaagaatggagatatgcattcaagagaggtgctatcacatagatctagtctgaagtctggaacactttcctcttctatgacccctctctccccagtattatcttacttgcaaaatggagaccaaattctatcctgtgaggcttttaattgcaccatagtatgctctgagtagctttacactgcctggtactgatagtagtggctcgatttttaagagccttcaattgtagatgaacatctctgttatttatccctcattcatccatccgttcattcattcagccttcaatcaacatctcttgagtgtctattatgtacaggacatgtactgagacaaaaaggaaacataagagctttttcactctaaaaatcttggcaataatgtcaacaccagaaagcctcctctggagaatcttacagagtgattgtagtttaatacaggaacacacagggctgtgtagcatgataccaggcccaggagatcagtaattacaaattaagggttaaatcagagattattcaacagagagggagaaaggaggagacagagggaggacctgttgtgttccagccattctggtattcctttatgtatctaatttcattcaaacctcacaacagtcttgtgaggcccttatataattactcccattttgcagatgaagtaactgaggcttagaaaggttaatagcaccggggaacaatttctctgggtgagaattgggactctgttgctggtcttctcagttcatttcctgaggtggatttactgagagaaggtgaaataaagccatatttagtataccagagaaggtagattttaagaatggtctcagtgttaatactgagaaaaagtcctgtcagttcagaaaaaatgtgaagtctactttagtattcctgtaatactaaaccgttgagtttctaaatatttatttattctaacaaaaagcaattactacaaatggatgacacatttaatgaacacaattttattttttttctgtaactgtgcttgttgaatgtcaatcatatttaaagggaatgactttgaagtaaaaccttttttcttgctactgaaaaaaatggagttgttttgggtggtaaagtgttaaggaatagggacagctggtcacacaaggaactcttgaaggccacatgtgaaaacctgtcacttgcacagaggccagtcccactaaggtgaccagagtgggctccaagcacaaactgccattggctatagatgggactgtgtccccccaaaattcatgtgttggagccttaaccctcaatgtgatggtatttgagatggggcctttggtaagggaagtttagatgaggtcacgagggtaggaccctcatgatgggatgagtccccttacaagacctctggcttgggccgggcgtggtggctcacacctgtaatcccaacactttgggaggccaaggcaggtagatcacttgatgccaggagttccagaccaggctggccgacatggtgaaaccccatctctactaaaaaatataaaaattagccgggctttgtggcatgtgcctgtaatcccagctatttggcaggctgaggcatgagaatcgcttgaacccaggaggtggaggttacagtgagctgagagtgccccactgcactccagcctgggtgacagagcgagactttgtcccaaaacaaaataggtgaggggatagcgaatgcactcagggtcagcagtggagtttaaaaattgtctcttttcaacttatttaaatgacagcacctgagaagaggaaccgttttacactggatgtttctcatgtagaacaagaaatctttctggaattgatgtttacatgtctgttgttggtcatctctcctgtgtcttaaatactttaatgttggaagagcatagtgtttgggctagtgggtttctgacagcccatgggaatgccctgaaactactgtatctgatgtttgttttcgatgaggttccatgttttgttttcttgggaataaattaatatattgttttccaaaaaaaaaaaaaaaaaaaa
