# Supplementary material for: Inhibition of miR-199b-5p reduces pathological alterations in osteoarthritis by potentially targeting Fzd6 and Gcnt2
Source: eLife. 2024 May 21;12:RP92645. doi: 10.7554/eLife.92645 (PMC11108644; doi:10.7554/eLife.92645)
Supplement: Supplementary file 1. [file elife-92645-supp1.docx]

**Supplement table 1: Primer List**

| **gene** | **sequence** |
| --- | --- |
| U6 | GSP:5’GCTTCGGCAGCACATATACTAAAAT3’  R:5’CGCTTCACGAATTTGCGTGTCAT3’ |
| hsa-miR-199b-5p | GSP:5’GGGACCCCAGTGTTTAGACTAT3’  R: 5'GTGCGTGTCGTGGAGTCG3’ |
| hsa-miR-338-3p  hsa-miR-15b-3p  hsa-miR-1296-5p  hsa-miR-3168 | GSP:5'GGGGGTCCAGCATCAGTGA3’  R:5’GTGCGTGTCGTGGAGTCG3’  GSP:5'GGGGCGAATCATTATTTGCT3’  R:5’ GTGCGTGTCGTGGAGTCG3’  GSP:5’GGTATTAGGGCCCTGGCTC3’  R:5’GTGCGTGTCGTGGAGTCG3’  GSP:5’TTGGGGGGGAGTTCTACAG3’  R:5’GTGCGTGTCGTGGAGTCG3’ |
| mmu-miR-199b-5p | GSP:5’GGGACCCCAGTGTTTAGACTAT3’  R: 5'GTGCGTGTCGTGGAGTCG3’ |
| mmu-miR-15b-3p | GSP:5'GGGGCGAATCATTATTTGCT3’  R:5’ GTGCGTGTCGTGGAGTCG3 |
| mmu-miR-338-3p | GSP:5'GGGGGTCCAGCATCAGTGA3’  R:5’GTGCGTGTCGTGGAGTCG3’ |
| Gapdh | F: TGTTTCCTCGTCCCGTAGA  R: ATCTCCACTTTGCCACTGC |
| Hif1α  Fzd6  Gcnt2  Myh9  Caprin1  Atg14  Mmp3  Adamts5  Aggrecan  Sox9  Col2a1 | F: GGGGAGGACGATGAACATCAA  R: GGGTGGTTTCTTGTACCCACA  F: AGAGGTGAAAGCGGACGGA  R: AGAGAGTCTGGAGATGGATGCT  F: TCCTGGACGGGTAACCTCAG  R: CTGCAAGTCTCCGTTTCCATAG  F: AGAAGTTGGTATGGGTGCCTT  R: CCCTGAGTAGTATCGCTCCTTG  F: GAAGCAGATTCTCGGCGTAAT  R: TCCCCTTTATTCATTCGTTCCTG  F: GAGGGCCTTTACGTGGCTG  R: AATAGACGAAATCACCGCTCTG  F: ACTGTGTCCCAAGGAGAGGAG  R: AAACCATCTACACAGTTCAGACAC  F: ATGCAGCCATCCTGTTCACC  R: AAGGCCAAGTAGATGCCCAATTT  F: CACTGTCAAAGCACCATGCC  R: TAGGCTGGCTCCCATTCAGT  F: TAATTCCCCAGGCTCTTGGAT  R: GCAGCCGGGATTTAAGGCTC  F: CACGCATGAGCCGAAGCTA  R: GGGTTTCCACGTCTCACCA |

**Supplement table 2: Basic information for recruiting patients**

| Baseline | KOA（N=15） | HC（N=10） | *P* value |
| --- | --- | --- | --- |
| Age | 52.67 ± 7.35 | 51.50 ± 7.71 | 0.706 |
| Height | 1.63±0.07 | 1.60±0.05 | 0.201 |
| Weight | 65.03±9.53 | 61.30±8.47 | 0.327 |
| BMI | 24.28 ± 2.39 | 23.90± 2.65 | 0.715 |
